# Feasibility of Videoconference-Based Cognitive Behavioral Therapy for Somatic Symptom Disorder: Single-Arm Pilot Trial

**DOI:** 10.2196/86698

**Published:** 2026-05-20

**Authors:** Hideki Nakamura, Kayoko Taguchi, Masayuki Katsushima, Sho Okawa, Mirai Miyoshi, Eiji Shimizu

**Affiliations:** 1 Department of Mental Health Nursing, Faculty of Nursing, Toho University, 4-16-20, Omori-Nishi, Ota-ku, Tokyo, 143-0015, Japan, 81 03-3762-9881; 2 Research Center for Child Mental Development, Chiba University, Chiba, Japan; 3 Department of Rehabilitation, Faculty of Health Care and Medical Sports, Teikyo Heisei University, Ichihara, Japan; 4 Department of Life Sciences, The University of Tokyo, Tokyo, Japan; 5 Department of Cognitive Behavioral Physiology, Graduate School of Medicine, Chiba University, Chiba, Japan

**Keywords:** somatic symptom disorder, videoconference-based cognitive behavioral therapy, cognitive behavioral therapy, feasibility, quality of life, telemedicine

## Abstract

**Background:**

Somatic symptom disorder (SSD) is a mental disorder marked by persistent somatic symptoms and maladaptive health-related thoughts, feelings, or behaviors. Cognitive behavioral therapy has been shown to be effective in treating SSD, reducing patients’ somatic symptoms, depressive symptoms, and anxiety symptoms. However, challenges remain—including limited access to treatment. Videoconference-based cognitive behavioral therapy (vCBT) has emerged as a promising approach, offering flexible and tailored treatment while addressing the shortage of medical resources and potentially reducing patient dropout.

**Objective:**

This study examined the feasibility of vCBT for patients with SSD and explored secondary outcomes related to the physical component of health-related quality of life (HRQOL) as assessed using the Medical Outcomes Study 36-Item Short Form Health Survey (SF-36) physical component summary (PCS) score and other exploratory clinical outcomes.

**Methods:**

This single-arm pilot trial evaluated feasibility as the primary outcome assessed via recruitment rate, retention rate, session completion rate, and safety. Ten participants with SSD were enrolled, and all received 6 weekly 50-minute vCBT sessions. Secondary outcomes included physical HRQOL (SF-36 PCS) as the key secondary outcome, as well as mental HRQOL, depressive symptoms, somatic symptom severity, anxiety symptoms, health anxiety, pain, insomnia, and generic HRQOL. These outcomes were measured at the preintervention (week 0) and postintervention (week 6) time points and 1-month follow-up (week 10) and were analyzed using 2-sided paired *t* tests.

**Results:**

All feasibility criteria were met, with a recruitment rate of 83.3% (10/12), a retention rate of 100% (10/10), a session completion rate of 100% (10/10), and no adverse events reported. The SF-36 PCS score improved by 4.99 points at the postintervention time point, representing a large within-group effect (Cohen *d*=0.99, 95% CI 0.21‐1.74). Medium to large improvements were also observed in mental HRQOL, depressive symptoms, anxiety symptoms, health anxiety, pain, insomnia, and generic HRQOL, and most of these improvements were observed at the 1-month follow-up.

**Conclusions:**

Our findings indicate that vCBT is a feasible and safe approach for SSD, with findings suggesting acceptability and preliminary evidence of effectiveness.

## Introduction

### Background

Somatic symptom disorder (SSD) is defined in the *Diagnostic and Statistical Manual of Mental Disorders, Fifth Edition* (*DSM-5*), as one or more distressing somatic symptoms or bodily sensations accompanied by excessive thoughts, feelings, or behaviors related to the symptoms persisting for more than 6 months and causing significant disruption in daily life [1]. In the *DSM-5*, SSD replaced the former *Diagnostic and Statistical Manual of Mental Disorders, Fourth Edition*, category of “somatoform disorders,” with the key revision shifting the diagnostic focus from “medically unexplained symptoms” (MUS) to patients’ “excessive psychological and behavioral responses” to their symptoms [2]. Against this background, the prevalence of SSD has been estimated at 3% to 4% in the general population, with higher rates observed among women [3]. SSD is associated with severe functional impairment, reduced quality of life, and frequent comorbidity with anxiety and depression [4,5]. A recent meta-analysis estimated the global burden of somatoform disorders at 662.4 years lived with disability per 100,000 person-years (95% CI 350.2‐974.6). In terms of the mental disorders included in the Global Burden of Disease Study 2021, this estimate suggests that somatoform disorders could represent the second-highest disease burden after depressive disorders [4]. Faced with these difficulties, patients often pursue multiple treatments, yet satisfactory outcomes are uncommon. Such persistent treatment seeking may increase dissatisfaction with the health care system [6]. Moreover, excessive health care use by patients with SSD has been identified as a contributor to rising medical costs, posing a broader social concern [7,8]. Additionally, the long-term prognosis of SSD is generally poor, with follow-up studies reporting high disability rates [9]. Given this substantial burden, pharmacological treatments (eg, antidepressants) have sometimes been considered for SSD. However, such treatments show limited efficacy [10], and evidence of their effectiveness is insufficient; accordingly, they are rarely recommended as stand-alone interventions. Instead, pharmacotherapy is often used to manage comorbid symptoms, which can lead to polypharmacy and an increased risk of side effects [11].

The treatment guidelines for SSD published by the Association of the Scientific Medical Societies in Germany discourage the use of stand-alone drug therapy and advocate for combination with psychosocial interventions [12]. Among these, cognitive behavioral therapy (CBT) is the most commonly used psychosocial intervention for SSD [13,14]. CBT for SSD is grounded in a cognitive behavioral model [15]. Within this framework, Sharpe et al [16] proposed that SSD arises from the interaction of physical, cognitive, behavioral, emotional, and environmental factors, resulting in a self-perpetuating vicious cycle. This multidimensional model highlights the need for therapeutic approaches that address not only somatic symptoms but also patients’ cognitive and behavioral responses.

As SSD is a relatively new diagnosis, evidence from CBT studies directly targeting it remains limited. Therefore, research on somatoform disorders and MUS provides valuable evidence. CBT has been proven effective at improving physical functioning and reducing symptom severity for patients with MUS [17]. Similarly, a meta-analysis of 15 randomized trials involving a total of 1671 participants with somatoform disorders and MUS indicated that CBT significantly reduced depressive symptoms and anxiety symptoms as well as the severity of somatic symptoms [18]. Although conventional CBT is effective, its length and intensity can limit feasibility, particularly in primary care and resource-limited settings. The aforementioned meta-analysis of CBT for MUS reported a median of 8 sessions, and treatment compliance was significantly lower in interventions lasting longer than 12 weeks than in those lasting less than 12 weeks [17,18]. These findings suggest that shorter and less intensive programs may improve adherence and practicality. To address this, brief CBT programs have been developed and have shown promising acceptability and effectiveness [19,20]. For example, in the Netherlands, Sitnikova et al [21] implemented a 6-session brief CBT program delivered by local psychiatric nurses, which improved physical functioning and reduced functional impairment in patients with undifferentiated somatoform disorder.

Nevertheless, despite evidence supporting brief CBT, many patients still face barriers such as limited availability that prevent them from receiving treatment [22,23]. Against persistent barriers to accessing evidence-based care, digitally delivered psychotherapies for SSD have gained increasing attention. Self-guided internet-based cognitive behavioral therapy (iCBT) has demonstrated efficacy in reducing both somatic symptoms and illness anxiety in SSD and related functional somatic disorders compared with waitlist controls [24]. However, as iCBT relies largely on self-direction, it may be less suited to tailored case formulation and intervention strategies, particularly in heterogeneous and clinically complex presentations. To address these limitations, videoconference-based cognitive behavioral therapy (vCBT) has gained increasing attention. By enabling synchronous, real-time interaction with a therapist, vCBT facilitates the development of the therapeutic alliance and allows for ongoing treatment adjustment [25]. Moreover, vCBT has demonstrated outcomes comparable to those of face-to-face CBT across multiple conditions, including anxiety, depression, insomnia, and somatic symptoms [26,27].

Consistent with these findings, a large randomized controlled trial in individuals with high-impact chronic pain demonstrated that telephone-delivered CBT and vCBT yielded greater improvements in pain severity and functioning than iCBT [28]. Taken together, these findings suggest that vCBT may provide greater clinical benefits than iCBT for SSD.

However, to our knowledge, no study has yet examined vCBT specifically for SSD. Accordingly, further investigation is warranted to determine whether vCBT can address the unique barriers associated with SSD. Previous studies have highlighted several challenges in applying CBT to SSD, including limited clinical evidence, limited feasibility of long and intensive programs, and difficulties in accessing treatment. To address these issues, we considered that a vCBT program could offer an accessible and practical approach to psychological support for patients with SSD. vCBT remains a novel approach for SSD, and it is essential to examine feasibility before conducting large-scale trials. This pilot study provides preliminary evidence toward that goal.

### Objectives

This study examined the feasibility of vCBT for patients with SSD and explored secondary outcomes related to the physical component of health-related quality of life (HRQOL) and exploratory clinical outcomes. We hypothesized that vCBT would demonstrate high feasibility and safety and may lead to improvements in patients’ physical HRQOL and exploratory clinical outcomes.

## Methods

### Study Design

This study used a single-arm trial design to examine the feasibility of vCBT for SSD. To our knowledge, this study is the first to use vCBT as an intervention for SSD. Therefore, a single-arm trial focusing on feasibility rather than efficacy was deemed an appropriate design [29]. Feasibility criteria were set a priori with reference to pilot trial guidance, the CONSORT (Consolidated Standards of Reporting Trials) extension to randomized pilot and feasibility trials, and prior vCBT feasibility studies [30-32]. Thresholds included a recruitment rate of 70% or higher, retention rate of 80% or higher, session completion rate of 75% or higher, and safety defined as the absence of any serious adverse events.

Participants received vCBT for 6 weeks and were assessed at the preintervention (week 0) and postintervention (week 6) time points and 1-month follow-up (week 10).

### Ethical Considerations

This study was approved by the Ethics Committee of Chiba University School of Medicine (reference G2021007) and registered with the University Hospital Medical Information Network Clinical Trials Registry (UMIN000046138). The trial was prospectively registered prior to participant enrollment. All participants were thoroughly informed about the study through both written and verbal communication. Informed consent was obtained from all participants.

All study data were deidentified and managed using unique participant identifiers at the data center. Consent forms and related documents were securely stored in a locked cabinet.

Upon study completion, participants received a burden reduction fee of ¥5000 (approximately US $32) in the form of a gift card.

### Procedure

Participants were recruited from the Cognitive Behavioral Therapy Center, Chiba University Hospital, affiliated psychiatric clinics, and local psychiatric medical institutions via flyers. Screening procedures were conducted face-to-face by a psychiatrist using a structured interview to evaluate depressive symptoms and assess suicide risk. Suicide risk was further screened using item 9 of the Patient Health Questionnaire–9 (PHQ-9) [33]. Clinical interviews were conducted by trained nurses and occupational therapists to assess eligibility for participation based on patients’ backgrounds and the inclusion and exclusion criteria. Recruitment and intervention activities were conducted between September 2021 and March 2024.

### Eligibility Criteria

Inclusion criteria for this study were a primary diagnosis of SSD based on *DSM-5* criteria, age between 20 and 65 years, mental and physical capacity to understand and practice CBT for at least 2 continuous months, and access to and ability to use the internet and a computer to receive vCBT.

Exclusion criteria were *DSM-5* diagnoses of schizophrenia spectrum or bipolar disorders, intellectual disabilities, and autism spectrum disorders that could interfere with the intervention due to the exacerbation of symptoms and serious progressive physical illness. Additionally, individuals presenting with acute or severe depressive symptoms or elevated suicide risk were excluded from participation. Eligible participants were required to be under the care of a treating psychiatrist and demonstrate clinical stability, including stability of depressive symptoms. Patients whose SSD was predominantly characterized by chronic pain were excluded to focus on those with other primary somatic concerns. This approach was informed by prior evidence: the original protocol underpinning this program has already demonstrated efficacy in chronic pain populations [34]. Accordingly, to reduce sample heterogeneity and evaluate feasibility in SSD presentations characterized by nonpain symptoms, individuals whose predominant concern was chronic pain were excluded. All participants were screened using the Autism Spectrum Quotient total score (cutoff score <33) and the Japanese Adult Reading Test–estimated IQ (cutoff score ≥80). These cutoffs, based on validation studies, were used to screen for characteristics that could interfere with participation [35,36].

### Interventions

The vCBT program for SSD was developed by 3 researchers (HN, KT, and ES). Drawing from the 16-week CBT program for chronic pain by Taguchi et al [34], we set the total number of vCBT sessions to 6 to enhance feasibility and reduce participant burden. Sessions lasted 50 minutes each and were held once per week for 6 weeks. The program comprised 6 modules and incorporated principles of the fear-avoidance model (from chronic pain CBT) into the treatment for SSD. According to the fear-avoidance model, catastrophizing somatic symptoms leads to excessive focus on those symptoms, avoidance of even minor risks in an effort to ensure safety, and an increase in maladaptive safety behaviors (eg, avoidance) that reinforce the cycle of illness. Such behaviors can maintain or worsen somatic symptoms and create a self-perpetuating cycle that impairs daily functioning and mood [37]. The program was designed to address this vicious cycle of overemphasis on somatic symptoms and safety behaviors [16,38].

The program content included (1) introduction and assessment, (2) psychoeducation, (3) case formulation, (4) relaxation techniques, (5) analysis of safety behaviors, and (6) cognitive restructuring. The therapist and participant engaged in real-time remote sessions via a videoconferencing system, connecting the participant’s home with Chiba University Hospital for vCBT sessions. After each session, participants completed homework assignments to integrate the therapy strategies into their daily lives. Participants were provided with a study contact email address to use if they had questions about the session content or homework assignments.

### Therapist and Quality Control

vCBT was delivered by the first author, a nurse therapist who has received specialized training in CBT. The therapist completed a 2-year intensive CBT training course as part of the Chiba Improving Access to Psychological Therapies project [39]. All intervention sessions were recorded, and the therapist received monthly supervision from a psychiatrist (ES) specialized in CBT. To minimize bias, the therapist was not involved in outcome data entry. All self-reported assessments were collected and managed independently of intervention delivery.

### Outcomes

#### Primary Outcome: Feasibility

The primary outcome was feasibility. Feasibility was evaluated using 4 predefined indicators: recruitment rate, retention rate, session completion rate, and safety. The thresholds were a recruitment rate of at least 70%, a retention rate of at least 80%, a session completion rate of at least 75%, and no serious adverse events.

#### Secondary Outcomes: Exploratory Clinical Outcomes

Secondary outcomes were analyzed on an exploratory basis. The key secondary outcome was the Medical Outcomes Study 36-Item Short Form Health Survey (SF-36) physical component summary (PCS) score, whereas other secondary outcomes included mental HRQOL, depressive symptoms, somatic symptom severity, anxiety symptoms, health anxiety, pain, insomnia, and generic HRQOL.

##### Key Secondary Outcome

The key secondary outcome of this study was the change in the SF-36 PCS score from the pre- to postintervention time point. The SF-36 is a globally standardized self-administered scale for measuring HRQOL. It provides 2 norm-based summary scores: the PCS and the mental component summary (MCS). The PCS is a norm-based summary score, with higher scores indicating better physical HRQOL [40]. The Japanese version of the SF-36 was used in this study, and its reliability and validity have been previously established [41].

##### Other Secondary Outcomes

###### SF-36 MCS Score

The SF-36 MCS score was used to assess mental HRQOL. The MCS is a norm-based summary score, with higher scores indicating better mental HRQOL [40].

###### Patient Health Questionnaire–9

We used the PHQ-9 to assess depressive symptoms. The total score ranges from 0 to 27. A score of 0 to 4 indicates minimal depression, 5 to 9 indicates mild depression, 10 to 14 indicates moderate depression, 15 to 19 indicates moderately severe depression, and 20 to 27 indicates severe depression [42]. The Japanese version of the PHQ-9 was used in this study, and its reliability and validity have been previously established [43].

###### Patient Health Questionnaire–15

The Patient Health Questionnaire–15 (PHQ-15) is a 15-item self-administered instrument designed to assess somatic symptom severity. Item 4 evaluates menstrual problems and is applicable only to female respondents; therefore, the total score range differs by sex (0‐30 for female individuals and 0‐28 for male individuals) [44]. The Japanese version of the PHQ-15, which has established reliability and validity, was used in this study [45].

###### Generalized Anxiety Disorder–7

The Generalized Anxiety Disorder–7 (GAD-7) is a self-administered instrument designed to assess anxiety symptoms. It has 7 items scored as 0 (“not at all”), 1 (“several days”), 2 (“more than half the days”), and 3 (“nearly every day”). The total score ranges from 0 to 21. A score of 0 to 4 indicates minimal anxiety, 5 to 9 indicates mild anxiety, 10 to 14 indicates moderate anxiety, and 15 to 21 indicates severe anxiety [46]. The Japanese version of the GAD-7 was used in this study, and its reliability and validity have been previously established [47].

###### Short Health Anxiety Inventory

The Short Health Anxiety Inventory (SHAI) is an 18-item self-administered scale for measuring health anxiety, with each item scored from 0 to 3, resulting in a total score ranging from 0 to 54 [48]. The Japanese version of the SHAI was used in this study, and its reliability and validity have been previously demonstrated [49].

###### Brief Pain Inventory

The Brief Pain Inventory (BPI) is a brief self-administered questionnaire that measures the intensity of pain and its impact on daily life. It consists of 2 components: pain severity (4 items) and pain interference (7 items). Each of the items is rated on a scale from 0 to 10 [50]. In this study, an overall mean BPI score was calculated as the mean of all 11 items, ranging from 0 to 10, with higher scores indicating greater pain severity and interference. The Japanese version of the BPI was used in this study, and its reliability and validity have been established [51].

###### Insomnia Severity Index

The Insomnia Severity Index (ISI) is a 7-item self-report questionnaire for assessing the nature, severity, and impact of insomnia. Each item is rated on a scale from 0 to 4, yielding a maximum possible score of 28 [52]. The Japanese version of the ISI was used in this study, and its reliability and validity have been previously demonstrated [53].

###### EQ-5D-5L

The EQ-5D-5L is a comprehensive assessment scale developed to evaluate generic HRQOL. It measures current health status across 5 dimensions: mobility, self-care, usual activities, pain and discomfort, and anxiety and depression. Each dimension has 5 response levels [54]. The EQ-5D-5L index score was calculated using the Japanese value set [55].

### System Safety

This study used Microsoft Teams [56], a videoconferencing system with ISO/IEC 27001 certification. Microsoft Teams has implemented measures to prevent unauthorized access and data breaches, ensuring data protection and addressing potential security and privacy concerns.

### Safety Monitoring

Throughout the intervention period, suicidal ideation and worsening depressive symptoms were systematically assessed at each session. When risk was suspected, a psychiatrist conducted an additional evaluation, and a protocol was implemented to refer participants to psychiatric or emergency services as necessary. Additionally, to ensure comprehensive safety monitoring, all adverse events were reported irrespective of their relevance to the intervention.

### Statistical Analysis

This study was designed as a single-arm pilot trial. Statistical analyses were performed using SPSS Statistics (version 29.0; IBM Corp) following a prespecified analysis plan. Continuous variables are reported as means and SDs, and categorical variables are reported as counts and percentages. The normality of each continuous outcome was assessed using the Shapiro-Wilk test, and all variables met the assumption of normality, allowing for the use of 2-sided paired *t* tests for subsequent analyses.

For the key secondary outcome (SF-36 PCS), changes from the pre- to postintervention time points and follow-up were analyzed using paired *t* tests. For the other secondary outcomes, changes in SF-36 MCS, PHQ-9, PHQ-15, GAD-7, SHAI, BPI, ISI, and EQ-5D-5L scores from the pre- to postintervention time points and follow-up were analyzed. Effect sizes (Cohen *d*) were calculated for both pretest-posttest and pretest–follow-up comparisons of secondary outcomes, with the absolute values categorized as small (0.20), medium (0.50), and large (0.80) [57]. All tests were 2-sided with an α of .05. All analyses were conducted on an exploratory basis, and no adjustment for multiple comparisons was applied to either the key secondary outcome or the other secondary outcomes. We initially aimed to enroll 12 participants per the rule of thumb for pilot studies by Julious [58]. Due to recruitment constraints, 10 participants were enrolled.

## Results

### Recruitment

**Figure 1.**
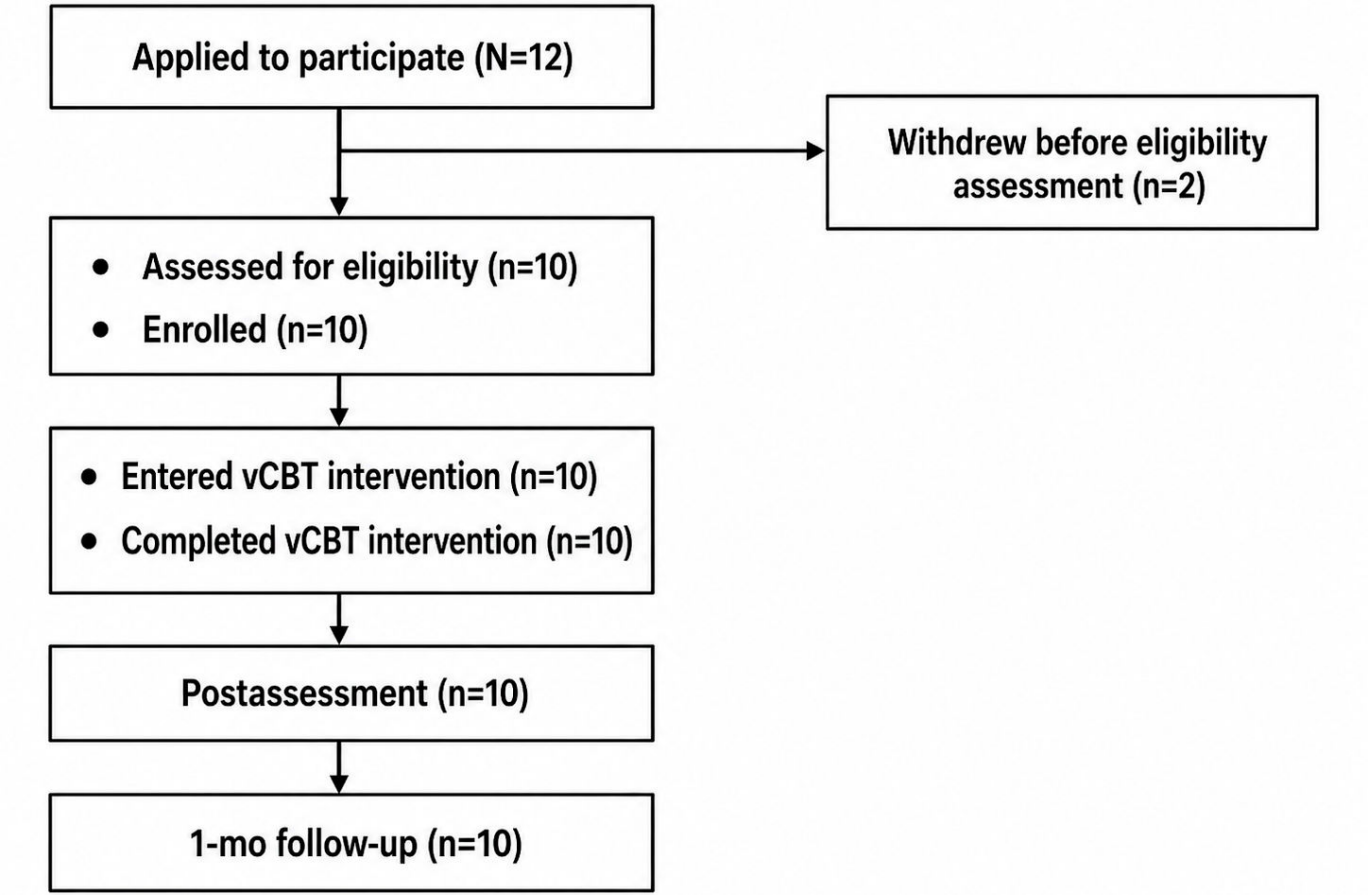
CONSORT (Consolidated Standards of Reporting Trials) flow diagram of participant recruitment and retention. A total of 12 individuals applied to participate, of whom 2 (16.7%) withdrew before the eligibility assessment. The remaining 10 participants were enrolled in the study and completed the vCBT intervention, postintervention assessment, and 1-month follow-up. vCBT: videoconference-based cognitive behavioral therapy.

Figure 1 shows the CONSORT flow diagram for this study. A total of 12 individuals applied to participate, but 2 (16.7%) withdrew before the eligibility assessment, leaving 10 (83.3%) participants who underwent eligibility assessment and were enrolled in the study. Although we targeted 12 participants, recruitment ended at 10 due to the fixed study time frame and institutional constraints. All enrolled participants completed the vCBT intervention, postintervention assessment, and 1-month follow-up.

### Demographic and Clinical Characteristics

Table 1 presents the demographic and clinical characteristics of the participants, including age, sex, psychotropic medication use, comorbid psychiatric disorders, principal somatic symptoms, years of education, total employment duration, number of cohabitants, estimated IQ, and AQ total score. Of the 10 participants, 5 (50%) were female, and 5 (50%) were male. The participants’ ages ranged from 20 to 52 years (mean 36.8, SD 11.0 years). At the time of consent, 50% (5/10) of the participants were employed. In total, 60% (6/10) of the participants had at least one comorbid psychiatric diagnosis in addition to SSD. The comorbid diagnoses included agoraphobia (n=3), panic disorder (n=1), generalized anxiety disorder (n=2), and major depressive disorder (n=1). A total of 80% (8/10) of the participants were taking at least one psychotropic medication, including benzodiazepine anxiolytics (n=4), antidepressants (n=4), antipsychotics (n=1), and mood stabilizers (n=1). The principal somatic symptom categories were fatigue (3/10, 30%), dizziness (3/10, 30%), numbness (2/10, 20%), and stomach discomfort (2/10, 20%).

**Table 1.** Characteristics of participants (N=10).

Characteristic	Values
Age (y), mean (SD)	36.8 (11.0)
Sex, n (%)	
Male	5 (50)
Female	5 (50)
Psychotropic medication use, n (%)	
Yes	8 (80)
No	2 (20)
Comorbid psychiatric disorders, n (%)^a^	6 (60)
No comorbid disorders (SSD^b^ only)	4 (40)
Major depressive disorder	1 (10)
Panic disorder	1 (10)
Agoraphobia	3 (30)
GAD^c^	2 (20)
Principal somatic symptoms, n (%)	
Fatigue	3 (30)
Dizziness	3 (30)
Numbness	2 (20)
Stomach discomfort	2 (20)
Education (y), mean (SD)	14.2 (2.4)
Total employment duration (y), mean (SD)	13.6 (12.1)
Number of cohabitants, mean (SD)	3.0 (1.3)
Estimated IQ via the JART^d^, mean (SD)	105.8 (6.5)
AQ^e^ total score (0-50), mean (SD)	17.9 (9.3)

a Participants could have more than one comorbid disorder.

b SSD: somatic symptom disorder.

c GAD: generalized anxiety disorder.

d JART: Japanese Adult Reading Test.

e AQ: Autism Spectrum Quotient.

Each participant was classified into a single principal somatic symptom category. None of the participants changed their medication during the study period, and none initiated any other psychotherapy. Because no outcome data were missing, the intention-to-treat and complete-case populations were identical; therefore, only intention-to-treat results are reported.

### Feasibility Results

With respect to feasibility, the recruitment rate was 83.3% (10/12), the retention rate was 100% (10/10), the session completion rate was 100% (10/10), and no adverse events were reported; thus, all predefined feasibility criteria were met. All sessions were delivered according to the treatment manual, with no major modifications or deviations.

### Exploratory Clinical Findings

#### Key Secondary Outcome

**Figure 2.**
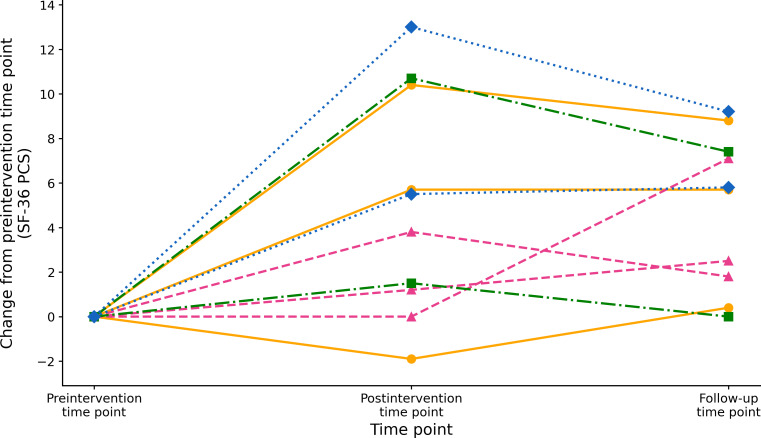
Individual changes in Medical Outcomes Study 36-Item Short Form Health Survey (SF-36) physical component summary (PCS) scores grouped by principal somatic symptom. Lines indicate individual changes from the preintervention time point (week 0) to the postintervention time point (week 6) and follow-up (week 10). Colors and markers represent symptom categories: fatigue (3/10, 30%) is represented by the orange solid lines, dizziness (3/10, 30%) is represented by the pink dashed lines, numbness (2/10, 20%) is represented by the green dashed-dotted lines, and stomach discomfort (2/10, 20%) is represented by the blue dotted lines. A value of 0 indicates no change from the preintervention time point.

The SF-36 PCS score showed a significant improvement from the pre- to postintervention time point, rising from 40.29 (SD 15.01) to 45.28 (SD 16.39), with a mean change of 4.99 (SD 5.02) points and a large effect size (*t*_9_=3.1; *P*=.01; *d*=0.99, 95% CI 0.21-1.74). Similarly, the SF-36 PCS score increased from 40.29 (SD 15.01) at the preintervention time point to 45.16 (SD 14.77) at follow-up, with a mean change of 4.87 (SD 3.43) points and a large effect size (*t*_9_=4.5; *P*=.002; *d*=1.42, 95% CI 0.51-2.30). Figure 2 shows participant-level trajectories in SF-36 PCS scores expressed as change from the preintervention time point, with lines grouped by principal somatic symptom at the pre- and postintervention time points and follow-up.

#### Other Secondary Outcomes

Tables2  3 present the means, SDs, and effect sizes for the other secondary outcomes at each time point. The SF-36 MCS score increased from 42.20 (SD 10.07) at the preintervention time point to 49.58 (SD 3.38) at the postintervention time point, a medium to large improvement (*t*_9_=2.5; *P*=.03; *d*=0.79). By follow-up, it rose to 50.05 (SD 5.99), with a large effect size (*t*_9_=2.9; *P*=.02; *d*=0.92).

**Table 2.** Preliminary effects of videoconference-based cognitive behavioral therapy on other secondary outcomes (N=10).

Outcome	Pre- to postintervention time point			Preintervention time point to follow-up		
	Change, mean (SD)	Cohen *d* (95% CI)	*P* value	Change, mean (SD)	Cohen *d* (95% CI)	*P* value
SF-36 MCS^a^	7.38 (9.29)	0.79 (0.06 to 1.50)	.03	7.85 (8.56)	0.92 (0.15 to 1.65)	.02
PHQ-9^b^	−4.40 (2.80)	−1.57 (−2.50 to −0.61)	.001	−5.10 (3.90)	−1.31 (−2.15 to −0.43)	.003
PHQ-15^c^	−1.90 (2.88)	−0.66 (−1.33 to 0.04)	.07	−2.50 (2.76)	−0.91 (−1.63 to −0.14)	.02
GAD-7^d^	−5.50 (2.95)	−1.86 (−2.89 to −0.80)	<.001	−6.00 (4.62)	−1.30 (−2.14 to −0.42)	.003
BPI^e^	−1.90 (1.33)	−1.43 (−2.31 to −0.51)	.001	−1.90 (1.85)	−1.03 (−1.79 to −0.23)	.01
SHAI^f^	−8.40 (4.03)	−2.08 (−3.20 to −0.94)	<.001	−11.50 (6.75)	−1.70 (−2.68 to −0.69)	<.001
ISI^g^	−2.50 (3.21)	−0.78 (−1.48 to −0.05)	.04	−3.30 (2.67)	−1.24 (−2.06 to −0.38)	.004
EQ-5D-5L index score	0.14 (0.14)	0.96 (0.19 to 1.71)	.01	0.15 (0.11)	1.26 (0.40 to 2.08)	.003

a SF-36 MCS: Medical Outcomes Study 36-Item Short Form Health Survey mental component summary.

b PHQ-9: Patient Health Questionnaire–9.

c PHQ-15: Patient Health Questionnaire–15.

d GAD-7: Generalized Anxiety Disorder–7.

e BPI: Brief Pain Inventory.

f SHAI: Short Health Anxiety Inventory.

g ISI: Insomnia Severity Index.

**Table 3.** Mean scores for the other secondary outcomes (N=10).

Outcome	Preintervention time point, mean (SD)	Postintervention time point, mean (SD)	Follow-up, mean (SD)
SF-36 MCS^a^ score	42.20 (10.07)	49.58 (3.38)	50.05 (5.99)
PHQ-9^b^ score (0 to 27)	13.00 (6.41)	8.60 (4.86)	7.90 (5.55)
PHQ-15^c^ score (0 to 30 for female individuals and 0 to 28 for male individuals)	13.60 (5.80)	11.70 (4.74)	11.10 (5.38)
GAD-7^d^ score (0 to 21)	12.70 (5.85)	7.20 (4.24)	6.70 (3.59)
BPI^e^ score (0 to 10)	4.74 (2.61)	2.84 (2.11)	2.84 (2.05)
SHAI^f^ score (0 to 54)	31.70 (9.90)	23.30 (8.54)	20.20 (9.19)
ISI^g^ score (0 to 28)	10.60 (4.35)	8.10 (5.67)	7.30 (5.17)
EQ-5D-5L index score (−0.025 to 1)	0.56 (0.24)	0.70 (0.23)	0.71 (0.21)

a SF-36 MCS: Medical Outcomes Study 36-Item Short Form Health Survey mental component summary.

b PHQ-9: Patient Health Questionnaire–9.

c PHQ-15: Patient Health Questionnaire–15.

d GAD-7: Generalized Anxiety Disorder–7.

e BPI: Brief Pain Inventory.

f SHAI: Short Health Anxiety Inventory.

g ISI: Insomnia Severity Index.

The mean PHQ-9 score dropped from 13.00 (SD 6.41) at the preintervention time point to 8.60 (SD 4.86) at the postintervention time point, a large reduction (*t*_9_=–5.0; *P*=.001; *d*=–1.57). By follow-up, it decreased further to 7.90 (SD 5.55), with a continued large effect size (*t*_9_=–4.1; *P*=.003; *d*=–1.31).

The mean PHQ-15 score went from 13.60 (SD 5.80) at the preintervention time point to 11.70 (SD 4.74) at the postintervention time point, a moderate but nonsignificant decrease (*t*_9_=–2.1; *P*=.07; *d*=–0.66). By follow-up, it was 11.10 (SD 5.38), indicating a large reduction (*t*_9_=–2.9; *P*=.02; *d*=–0.91).

The mean GAD-7 score showed a large reduction, from 12.70 (SD 5.85) at the preintervention time point to 7.20 (SD 4.24) at the postintervention time point (*t*_9_=–5.9; *P*<.001; *d*=–1.86). By follow-up, it was 6.70 (SD 3.59), indicating that the large improvement was maintained (*t*_9_=–4.1; *P*=.003; *d*=–1.30).

The mean BPI score showed a large reduction from 4.74 (SD 2.61) at the preintervention time point to 2.84 (SD 2.11) at the postintervention time point (*t*_9_=–4.5; *P*=.001; *d*=–1.43). At follow-up, it remained 2.84 (SD 2.05), indicating a sustained improvement (*t*_9_=–3.2; *P*=.01; *d*=–1.03).

The mean SHAI score showed a large reduction from 31.70 (SD 9.90) before the intervention to 23.30 (SD 8.54) after the intervention (*t*_9_=–6.6; *P*<.001; *d*=–2.08). By follow-up, it decreased further to 20.20 (SD 9.19), with a continued large effect size (*t*_9_=–5.4; *P*<.001; *d*=–1.70).

The mean ISI score showed a medium to large reduction from 10.60 (SD 4.35) at the preintervention time point to 8.10 (SD 5.67) at the postintervention time point (*t*_9_=–2.5; *P*=.04; *d*=–0.78). A similar reduction was observed from 10.60 (SD 4.35) before the intervention to 7.30 (SD 5.17) at follow-up (*t*_9_=–3.9; *P*=.004; *d*=–1.24).

The mean EQ-5D-5L index score showed a large improvement, increasing from 0.56 (SD 0.24) at the preintervention time point to 0.70 (SD 0.23) at the postintervention time point (*t*_9_=3.1; *P*=.01; *d*=0.96). By follow-up, it was 0.71 (SD 0.21), with a continued large effect size (*t*_9_=4.0; *P*=.003; *d*=1.26).

## Discussion

### Principal Findings

This study examined the feasibility of vCBT for SSD, with particular focus on physical HRQOL as the key secondary outcome. To the best of our knowledge, this is the first study to implement vCBT specifically for SSD. As hypothesized, there were no dropouts through the 1-month follow-up and no adverse events were reported, suggesting that vCBT is a feasible and potentially acceptable approach. Furthermore, within-group comparisons revealed medium to large effect size improvements in physical HRQOL, mental HRQOL, depressive symptoms, anxiety symptoms, health anxiety, pain, insomnia, and generic HRQOL, which were also observed at the 1-month follow-up. However, given the small sample size and limited statistical power, these results should be interpreted with caution.

All predefined feasibility criteria (recruitment, retention, session completion, and safety) were met in this study, and the dropout rate was 0%—an exceptionally low rate. This supports the feasibility of vCBT and suggests its acceptability. Previous CBT trials in SSD have reported dropout rates of approximately 10% to 23% [59,60]. Several factors may account for this difference with our study. First, the program was structured as six 50-minute sessions, representing a brief CBT format consistent with the framework recommended in primary care [61,62]. Brief interventions may help patients notice early changes, supporting motivation and lowering dropout risk. Indeed, a meta-analysis by Liu et al [18] found that shorter programs are associated with higher acceptability and completion rates—findings consistent with our results. Second, the videoconference format may have reduced barriers to accessing CBT by eliminating travel and waiting time [63,64]. Previous studies have noted that the impact of somatic symptoms and the resulting decline in HRQOL often make it difficult for patients to leave home for clinic visits, thereby hindering treatment continuation in SSD [65-67]. By removing these barriers while maintaining real-time therapist interaction and a strong therapeutic alliance, vCBT may have contributed to the zero dropout rate. Nevertheless, given this study’s pilot nature, any explanations for the low dropout rate must remain tentative.

The key secondary outcome—SF-36 PCS—showed a large improvement after the intervention (*d*=0.99). Consistent with prior research [21,59,60], this suggests that even brief CBT interventions can improve physical functioning. However, as this study had a small sample size and lacked a control group, caution is warranted in generalizing the results. The large effect size observed in this study may have been influenced by differences in participant characteristics compared with previous research [21]. Although direct comparison is difficult due to differences in study design, the mean age of the participants in this study was relatively young at 36.8 (SD 11.0) years, and the number and complexity of comorbid diagnoses may have been relatively limited. Indeed, prior research indicates that younger patients with fewer comorbidities may show greater treatment effects [67], which may have contributed to the improvements we observed.

When compared with the content of previous brief CBT programs for SSD and related somatoform disorders, our intervention shows several distinctive features. In the study by Sitnikova et al [21], a 6-session CBT program combined a results-focused model with problem-solving techniques, an approach widely used in Dutch primary care. That program emphasized building effective coping skills for daily challenges related to somatic symptoms, with problem-solving as a core element [68]. In contrast, our vCBT program emphasized reducing catastrophic thoughts and maladaptive safety behaviors [37,38]. We encouraged participants to view physical sensations as nonthreatening and curb excessive symptom focus and avoidance, with the goal of improving HRQOL without letting symptoms dominate their lives. This intervention may have helped reduce avoidance behaviors over the 6-week period, which may in turn have contributed to the gains in physical HRQOL. Beyond physical HRQOL, our findings suggest that vCBT may also enhance mental HRQOL, reduce pain, and improve generic HRQOL. These findings are broadly consistent with those of a previous meta-analysis [18]. Nevertheless, these findings are preliminary, and further studies are needed to clarify the mediating factors underlying such functional improvements.

This study has several strengths. First, the brief CBT format for SSD is pragmatic and easily implemented in primary care. Second, we comprehensively assessed multiple outcomes (physical and mental HRQOL, depressive symptoms, somatic symptom severity, anxiety symptoms, health anxiety, pain, insomnia, and generic HRQOL), and improvements were also observed at the 1-month follow-up despite the brief intervention. Third, the 100% session completion rate suggests that vCBT is a promising way to improve access for patients who have difficulty attending in-person sessions. Collectively, our findings provide preliminary evidence that vCBT is practical for patients with SSD and may be acceptable and that it may help alleviate barriers to care in routine clinical settings. In the future, larger randomized controlled trials with adequate power are needed to confirm vCBT’s efficacy and the durability of its effects.

### Limitations

This study has several limitations. First, although we observed improvements in physical HRQOL, the sample size was small, limiting statistical power. Furthermore, as this was a single-arm pilot trial with no control group, we cannot conclude that the outcomes were due solely to vCBT. Therefore, future studies should use adequately powered randomized controlled designs to determine clinically meaningful effects. Second, all outcome measures were based on patient self-report, introducing potential self-report bias. In addition, although the PHQ-15 scoring range differs by sex due to the inclusion of a menstrual item, the current analyses emphasized within-participant changes over time, thereby minimizing the impact of this difference. Future studies should consider using a sex-invariant approach or statistically adjusting for sex. Third, our participants were relatively young and presented with mild to moderate symptom burden and were recruited within a limited clinical network in the Tokyo metropolitan area, which limits generalizability to other populations, severity levels, or settings. Additionally, this sample had access to and the ability to use digital technology (prerequisites for vCBT), so the results may not extend to populations lacking such access or ability. Because individuals with predominantly chronic pain–related concerns were excluded, the generalizability of these findings may be limited in SSD presentations for which pain is the primary symptom. Fourth, we only evaluated vCBT’s effects up to the 1-month follow-up, so it remains unclear whether improvements persist beyond that point. Future research should examine the long-term sustainability of vCBT’s effects to address this limitation.

### Conclusions

This study suggests that vCBT is a feasible and safe treatment approach for SSD, with findings suggesting acceptability and preliminary evidence of effectiveness. vCBT may also provide a convenient option to improve access to care and enhance HRQOL. Future randomized controlled trials are needed to confirm its effectiveness and long-term benefits.

## Supplementary material

10.2196/86698 Checklist 1 CONSORT checklist.

## Abbreviations

BPI: Brief Pain Inventory
CBT: cognitive behavioral therapy
CONSORT: Consolidated Standards of Reporting Trials
DSM-5: Diagnostic and Statistical Manual of Mental Disorders, Fifth Edition
GAD-7: Generalized Anxiety Disorder–7
HRQOL: health-related quality of life
iCBT: internet-based cognitive behavioral therapy
ISI: Insomnia Severity Index
MCS: mental component summary
MUS: medically unexplained symptoms
PCS: physical component summary
PHQ-15: Patient Health Questionnaire–15
PHQ-9: Patient Health Questionnaire–9
SF-36: Medical Outcomes Study 36-Item Short Form Health Survey
SHAI: Short Health Anxiety Inventory
SSD: somatic symptom disorder
vCBT: videoconference-based cognitive behavioral therapy

## References

[1] (2013). Diagnostic and Statistical Manual of Mental Disorders.

[2] Sauer KS, Witthöft M, Rief W (2023). Somatic symptom disorder and health anxiety: assessment and management. Neurol Clin.

[3] Gureje O, De Guzman ML, Khaled SM (2025). The epidemiology of ICD-11 bodily distress disorder and DSM-5 somatic symptom disorder in new large-scale population surveys within the World Mental Health Survey Initiative. World Psychiatry.

[4] Siig EJ, Hvidtfelt Lykke VM, Mantilla Herrera AM, Jørgensen TS, Ferrari AJ, Santomauro DF (2025). The prevalence and estimated burden of somatoform disorders: a systematic review and meta-analysis of their epidemiology. Lancet Psychiatry.

[5] Löwe B, Levenson J, Depping M (2022). Somatic symptom disorder: a scoping review on the empirical evidence of a new diagnosis. Psychol Med.

[6] Shillaker J, Gibson C, Churchill J (2024). Healthcare experiences of people living with medically unexplained symptoms: a systematic review. Br J Nurs.

[7] Seo JH, Han M, Kang S, Kim SJ, Jung I, Kang JI (2024). Healthcare utilization and costs in patients with somatic symptom and related disorders compared with those with depression and healthy controls: a nationwide cohort study. Depress Anxiety.

[8] Jadhakhan F, Romeu D, Lindner O, Blakemore A, Guthrie E (2022). Prevalence of medically unexplained symptoms in adults who are high users of healthcare services and magnitude of associated costs: a systematic review. BMJ Open.

[9] Rask MT, Rosendal M, Fenger-Grøn M, Bro F, Ørnbøl E, Fink P (2015). Sick leave and work disability in primary care patients with recent-onset multiple medically unexplained symptoms and persistent somatoform disorders: a 10-year follow-up of the FIP study. Gen Hosp Psychiatry.

[10] Kleinstäuber M, Witthöft M, Steffanowski A, van Marwijk H, Hiller W, Lambert MJ (2014). Pharmacological interventions for somatoform disorders in adults. Cochrane Database Syst Rev.

[11] Wu CS, Chen TT, Liao SC, Huang WC, Huang WL (2024). Clinical outcomes, medical costs, and medication usage patterns of different somatic symptom disorders and functional somatic syndromes: a population-based study in Taiwan. Psychol Med.

[12] Schaefert R, Hausteiner-Wiehle C, Häuser W, Ronel J, Herrmann M, Henningsen P (2012). Non-specific, functional, and somatoform bodily complaints. Dtsch Arztebl Int.

[13] Maas Genannt Bermpohl F, Martin A (2025). Efficacy of cognitive behavioral therapy and acceptance- and mindfulness-based treatments in adults with bodily distress: a network meta-analysis. Psychother Psychosom.

[14] van Dessel N, den Boeft M, van der Wouden JC (2014). Non-pharmacological interventions for somatoform disorders and medically unexplained physical symptoms (MUPS) in adults. Cochrane Database Syst Rev.

[15] Sahm AH, Witthöft M, Bailer J, Mier D (2024). Putting the vicious cycle to the test: evidence for the cognitive behavioral model of persistent somatic symptoms from an online study. Psychosom Med.

[16] Sharpe M, Peveler R, Mayou R (1992). The psychological treatment of patients with functional somatic symptoms: a practical guide. J Psychosom Res.

[17] Menon V, Rajan TM, Kuppili PP, Sarkar S (2017). Cognitive behavior therapy for medically unexplained symptoms: a systematic review and meta-analysis of published controlled trials. Indian J Psychol Med.

[18] Liu J, Gill NS, Teodorczuk A, Li ZJ, Sun J (2019). The efficacy of cognitive behavioural therapy in somatoform disorders and medically unexplained physical symptoms: a meta-analysis of randomized controlled trials. J Affect Disord.

[19] Sumathipala A, Siribaddana S, Abeysingha MR (2008). Cognitive-behavioural therapy v. structured care for medically unexplained symptoms: randomised controlled trial. Br J Psychiatry.

[20] Burton C, Weller D, Marsden W, Worth A, Sharpe M (2012). A primary care Symptoms Clinic for patients with medically unexplained symptoms: pilot randomised trial. BMJ Open.

[21] Sitnikova K, Leone SS, van Marwijk HW, Twisk J, van der Horst HE, van der Wouden JC (2019). Effectiveness of a cognitive behavioural intervention for patients with undifferentiated somatoform disorder: results from the CIPRUS cluster randomized controlled trial in primary care. J Psychosom Res.

[22] Geraghty K, Scott MJ (2020). Treating medically unexplained symptoms via improving access to psychological therapy (IAPT): major limitations identified. BMC Psychol.

[23] Hanssen DJ, Bos LR, Finch TL, Rosmalen JG (2021). Barriers and facilitators to implementing interventions for medically unexplained symptoms in primary and secondary care: a systematic review. Gen Hosp Psychiatry.

[24] Liu S, Li Y, He Z, Sima X, Li J (2026). Efficacy of internet-based cognitive behavioral therapy on somatic symptom disorder and common related functional disorders: a meta-analysis of randomized controlled trials. Gen Hosp Psychiatry.

[25] Aafjes-van Doorn K, Spina DS, Horne SJ, Békés V (2024). The association between quality of therapeutic alliance and treatment outcomes in teletherapy: a systematic review and meta-analysis. Clin Psychol Rev.

[26] Ebrahimjee A, Hodsoll J, Valmaggia LR, Hickling LM, Riches S (2024). Video call-based cognitive behaviour therapy for adults with common mental health conditions: a systematic review and meta-analysis. Cogn Behav Ther.

[27] Matsumoto K, Hamatani S, Shimizu E (2021). Effectiveness of videoconference-delivered cognitive behavioral therapy for adults with psychiatric disorders: systematic and meta-analytic review. J Med Internet Res.

[28] DeBar LL, Mayhew M, Wellman RD (2025). Telehealth and online cognitive behavioral therapy-based treatments for high-impact chronic pain: a randomized clinical trial. JAMA.

[29] Lancaster GA, Thabane L (2019). Guidelines for reporting non-randomised pilot and feasibility studies. Pilot Feasibility Stud.

[30] Thabane L, Ma J, Chu R (2010). A tutorial on pilot studies: the what, why and how. BMC Med Res Methodol.

[31] Eldridge SM, Chan CL, Campbell MJ (2016). CONSORT 2010 statement: extension to randomised pilot and feasibility trials. BMJ.

[32] Matsumoto K, Sutoh C, Asano K (2018). Internet-based cognitive behavioral therapy with real-time therapist support via videoconference for patients with obsessive-compulsive disorder, panic disorder, and social anxiety disorder: pilot single-arm trial. J Med Internet Res.

[33] Simon GE, Rutter CM, Peterson D (2013). Does response on the PHQ-9 Depression Questionnaire predict subsequent suicide attempt or suicide death?. Psychiatr Serv.

[34] Taguchi K, Numata N, Takanashi R (2021). Clinical effectiveness and cost-effectiveness of videoconference-based integrated cognitive behavioral therapy for chronic pain: randomized controlled trial. J Med Internet Res.

[35] Wakabayashi A, Tojo Y, Baron-Cohen S, Wheelwright S (2004). The Autism-Spectrum Quotient (AQ) Japanese version: evidence from high-functioning clinical group and normal adults [Article in Japanese]. Shinrigaku Kenkyu.

[36] Matsuoka K, Uno M, Kasai K, Koyama K, Kim Y (2006). Estimation of premorbid IQ in individuals with Alzheimer’s disease using Japanese ideographic script (Kanji) compound words: Japanese version of National Adult Reading Test. Psychiatry Clin Neurosci.

[37] Crombez G, Eccleston C, Van Damme S, Vlaeyen JW, Karoly P (2012). Fear-avoidance model of chronic pain: the next generation. Clin J Pain.

[38] Leeuw M, Goossens ME, Linton SJ, Crombez G, Boersma K, Vlaeyen JW (2007). The fear-avoidance model of musculoskeletal pain: current state of scientific evidence. J Behav Med.

[39] Kobori O, Nakazato M, Yoshinaga N (2014). Transporting cognitive behavioral therapy (CBT) and the Improving Access to Psychological Therapies (IAPT) project to Japan: preliminary observations and service evaluation in Chiba. J Ment Health Train Educ Pract.

[40] Ware JE, Sherbourne CD (1992). The MOS 36-item short-form health survey (SF-36). I. Conceptual framework and item selection. Med Care.

[41] Fukuhara S, Ware JE, Kosinski M, Wada S, Gandek B (1998). Psychometric and clinical tests of validity of the Japanese SF-36 Health Survey. J Clin Epidemiol.

[42] Kroenke K, Spitzer RL, Williams JB (2001). The PHQ-9: validity of a brief depression severity measure. J Gen Intern Med.

[43] Muramatsu K, Miyaoka H, Kamijima K (2018). Performance of the Japanese version of the Patient Health Questionnaire-9 (J-PHQ-9) for depression in primary care. Gen Hosp Psychiatry.

[44] Kroenke K, Spitzer RL, Williams JB (2002). The PHQ-15: validity of a new measure for evaluating the severity of somatic symptoms. Psychosom Med.

[45] Muramatsu K (2014). An up-to-date letter in the Japanese version of PHQ, PHQ-9, PHQ-15. Niigata Seiryo Univ Clin Psychol Res.

[46] Spitzer RL, Kroenke K, Williams JB, Löwe B (2006). A brief measure for assessing generalized anxiety disorder: the GAD-7. Arch Intern Med.

[47] Muramatsu K, Miyaoka H, Kamijima K (2010). Examination of the validity and the utility of the Japanese version of the GAD‐7. Jpn J Psychosom Med.

[48] Salkovskis PM, Rimes KA, Warwick HM, Clark DM (2002). The Health Anxiety Inventory: development and validation of scales for the measurement of health anxiety and hypochondriasis. Psychol Med.

[49] Yamauchi G, Matsuoka H, Himachi M, Sasagawa S, Sakano Y (2009). Development and validation of Japanese version of the Short Health Anxiety Inventory. Jpn J Psychosom Med.

[50] Cleeland CS, Ryan KM (1994). Pain assessment: global use of the Brief Pain Inventory. Ann Acad Med Singap.

[51] Uki J, Mendoza T, Cleeland CS, Nakamura Y, Takeda F (1998). A brief cancer pain assessment tool in Japanese: the utility of the Japanese Brief Pain Inventory--BPI-J. J Pain Symptom Manage.

[52] Bastien CH, Vallières A, Morin CM (2001). Validation of the Insomnia Severity Index as an outcome measure for insomnia research. Sleep Med.

[53] Munezawa T, Morin CM, Inoue Y, Nedate K (2009). Development of the Japanese version of the Insomnia Severity Index (ISI-J). Jpn J Psychiatr Treat.

[54] Feng YS, Kohlmann T, Janssen MF, Buchholz I (2021). Psychometric properties of the EQ-5D-5L: a systematic review of the literature. Qual Life Res.

[55] Shiroiwa T, Ikeda S, Noto S (2016). Comparison of value set based on DCE and/or TTO data: scoring for EQ-5D-5L health states in Japan. Value Health.

[56] ISO/IEC 27001:2013 information security management standards. Microsoft.

[57] Cohen J (1988). Statistical Power Analysis for the Behavioral Sciences.

[58] Julious SA (2005). Sample size of 12 per group rule of thumb for a pilot study. Pharm Stat.

[59] Jongsma K, Darboh BS, Davis S, MacKillop E (2023). A cognitive behavioural group treatment for somatic symptom disorder: a pilot study. BMC Psychiatry.

[60] Luo J, Wang PC, Meng FQ (2025). Cognitive-behavioral therapy for patients with somatoform disorders: a pilot preliminary randomized controlled trial. Psychother Res.

[61] Mignogna J, Hundt NE, Kauth MR (2014). Implementing brief cognitive behavioral therapy in primary care: a pilot study. Transl Behav Med.

[62] Wortman MS, Lucassen PL, van Ravesteijn HJ (2016). Brief multimodal psychosomatic therapy in patients with medically unexplained symptoms: feasibility and treatment effects. Fam Pract.

[63] Fletcher TL, Hogan JB, Keegan F (2018). Recent advances in delivering mental health treatment via video to home. Curr Psychiatry Rep.

[64] Greenup EP, Best D (2025). Systematic review and meta-analysis of no show or non-attendance rates among telehealth and in-person models of care. BMC Health Serv Res.

[65] Benraad CE, Hilderink PH, van Driel D (2013). Physical functioning in older persons with somatoform disorders: a pilot study. J Am Med Dir Assoc.

[66] Liao SC, Ma HM, Lin YL, Huang WL (2019). Functioning and quality of life in patients with somatic symptom disorder: the association with comorbid depression. Compr Psychiatry.

[67] Sarter L, Heider J, Witthöft M, Rief W, Kleinstäuber M (2022). Using clinical patient characteristics to predict treatment outcome of cognitive behavior therapies for individuals with medically unexplained symptoms: a systematic review and meta-analysis. Gen Hosp Psychiatry.

[68] Zonneveld LN, van Rood YR, Timman R, Kooiman CG, Van’t Spijker A, Busschbach JJ (2012). Effective group training for patients with unexplained physical symptoms: a randomized controlled trial with a non-randomized one-year follow-up. PLoS One.

